# Relationship between Serum Transforming Growth Factor **β**1 Concentrations and the Duration of Type 1 Diabetes Mellitus in Children and Adolescents

**DOI:** 10.1155/2013/849457

**Published:** 2013-10-09

**Authors:** Katarzyna Zorena, Dorota Raczyńska, Piotr Wiśniewski, Ewa Malinowska, Małgorzata Myśliwiec, Krystyna Raczyńska, Dominik Rachoń

**Affiliations:** ^1^Department of Clinical and Experimental Endocrinology, Institute of Maritime and Tropical Medicine, Medical University of Gdańsk, 81-519 Gdynia, Poland; ^2^Department of Anesthesiology and Intensive Care Medicine, Medical University of Gdańsk, Poland; ^3^Department and Clinic of Ophthalmology, Medical University of Gdańsk, Poland; ^4^Department of Endocrinology and Internal Medicine, Medical University of Gdańsk, Poland; ^5^Department of Immunology, Medical University of Gdańsk, Poland; ^6^Department and Clinic of Paediatrics, Diabetology and Endocrinology, Medical University of Gdańsk, Poland

## Abstract

The aim of this study was to evaluate the relationship between serum transforming growth factor **β**1 (TGF-**β**1) concentrations and the duration of type 1 diabetes mellitus (T1DM) in children and adolescents. One hundred and sixteen patients with T1DM and 19 healthy controls were examined. Serum TGF-**β**1 concentrations were measured using the cytometric bead array (CBA). A positive association between the time of diabetes duration and higher serum TGF-**β**1 concentrations was observed. Similarly, the prevalence of microvascular complications, such as retinopathy and nephropathy, increased with the duration of diabetes. Logistic regression analysis showed that serum TGF-**β**1 concentrations and the duration of the disease are independent risk factors of microangiopathy development. Higher serum TGF-**β**1 concentrations were associated with a significant risk of microangiopathy development after 10 years of T1DM duration. In the successive years of the disease, the effect was even stronger. The results of our study indicate that serum TGF-**β**1 concentrations are one of the factors that may have an impact on the progression of vascular complications in children and adolescents with T1DM.

## 1. Introduction

Along with the increase in the prevalence of type 1 diabetes mellitus (T1DM) the number of patients with chronic vascular complications will rise significantly [1–3]. One of the most specific chronic diabetic complications is diabetic microangiopathy [3–6]. Clinically, microangiopathy manifests as retinopathy, nephropathy, and diabetic neuropathy—somatic as well as autonomic [4–6]. Many trials have also shown that the main determinants of the vessel damage in the course of diabetes are hyperglycemia and diabetes duration [7–9]. Diabetic microangiopathy means worsening of the quality of life and causing disability in patients with T1DM [10, 11]. It is particularly harmful when it concerns the youngest population, which often becomes debilitated already at the beginning of the adulthood [11]. Moreover, vascular complications are also seen in children and adolescents with T1DM who suffer for more than 5 years, and the progression in this group is often more rapid than in adults [3, 12, 13]. In the Oxford Regional Prospective Study (ORPS), it has been shown that about 25% of children with T1DM develop diabetic retinopathy within 5 years of the disease duration, but already as many as 60% and 80% after 10 years and 15 years, respectively [13]. The results from our studies have also shown that after 5 years of diabetes duration, albuminuria was detected in 29% and nonproliferative retinopathy in 27% of children and adolescents with T1DM [12, 14]. In the same study we have demonstrated that apart from the duration of diabetes, main factors influencing the development and progression of chronic vascular complications in children and adolescents with T1DM are growth factors, including vascular endothelial growth factor (VEGF) and angiogenin [12]. However, TGF-*β*1 has not yet been studied in this context. TGF-*β*1 belongs to a group of factors responsible for growth, differentiation and migration of cells, creation and degradation of the extracellular matrix, and apoptosis [15, 16]. In addition, it stimulates the formation of blood vessels and participates in wound healing and repair by increasing the production of extracellular matrix proteins [17]. However, its adverse activity has been shown in breast cancer, myocardial infarction, rheumatoid arthritis, osteoporosis, diabetic nephropathy, and retinopathy [17–19]. This cytokine is present in five different isoforms, three of which, that is, TGF-*β*1, TGF-*β*2, and TGF-*β*3, are coded by different genes. Out of these the best known is TGF-*β*1, produced by dendritic cells, leukocytes, and NK cells [15]. Despite extensive research on biology, genetics, and function of TGF-*β*1, there are few reports on this cytokine in children and adolescents with T1DM. Therefore, we have assumed that it would be worth evaluating serum levels of TGF-*β*1 in different groups of children and adolescents, depending on the duration of T1DM and the presence of vascular complications. Thus, the aim of our study was to evaluate the relationship between serum TGF-*β*1 levels and T1DM duration in children and adolescents with T1DM with and without vascular complications.

## 2. Subjects and Methods

### 2.1. Studied Subjects

One hundred and sixteen patients with T1DM aged 13.3 ± 3.9 years with diabetes duration of 9.7 ± 3.6 years from the Department and Clinic of Pediatrics, Diabetology and Endocrinology, Medical University of Gdańsk, were enrolled into the study. Diabetes was diagnosed according to the Polish Diabetes Association guidelines, which correspond with the guidelines of the American Diabetes Association [20, 21]. 

The inclusion criteria for children and adolescents into the group of patients with T1DM were age less then 21 years, diabetes duration ≥2 years, normal arterial pressure (systolic and diastolic), and no other chronic diseases. None of the diabetic patients were taking medications other than daily doses of insulin (0.83 ± 0.21 IU of insulin per day/kg of body weight). Blood pressure was measured using a 24 h blood pressure monitoring (ABPM) method. Various sizes of the cuff were used according to age, weight, and arm circumference of the studied subjects. All the ABPM reports which had less than 80% of technically correct measurements were excluded from the study. Arterial hypertension was diagnosed when mean ABPM values were above the 95th centile for the corresponding age, gender, and height [22]. Ophthalmologic examination was also performed. This included visual acuity tests, intraocular pressure and anterior segment estimation using the slit lamp (Topcon SL-82, Japan). After local administration of tropicamide (1% solution), the eye fundus was examined using the +90 D lens (Ocular Instruments, USA). A digital camera (Topcon Imaginet 2000, Japan) was used for the fluorescein angiography. The stage of retinopathy was diagnosed according to the guidelines of the International Diabetic Retinopathy Division [23]. Twenty-four-hour urine collection was performed three times during the period of 6 months for the evaluation of the daily albumin excretion. The urinary albumin was measured with immunoturbidimetric assay using a Tina-quant kit (Boehringer Mannheim GmbH, Germany). Albuminuria was diagnosed when at least two out of three urine samples displayed daily albumin excretion of between 30 and 299 mg/24 h, collected within 6 months from patients with well-controlled diabetes with no clinical or laboratory signs of ketoacidosis. Glycated haemoglobin (HbA1c) was measured with an immunoturbidometric method using a Unimate 3 set (Hoffmann-La Roche AG, Basel, Switzerland). The normal range was 4.0–6.0%. 

One hundred and sixteen patients with T1DM were examined and divided into four groups based on the duration of diabetes. The control group consisted of 19 healthy children and adolescents (11 boys and 8 girls, age range 6–18 yrs). Written informed consent was obtained from all the participants in the study, or from their parents or guardians. This study was approved by the Ethics Committee of The Medical University of Gdańsk, and the investigation was carried out in accordance with the principles of the Declaration of Helsinki as revised in 1996.

### 2.2. Serum TGF-*β*1 Measurements

The serum concentrations of TGF-*β*1 were measured using the cytometric bead array (CBA) as instructed by the manufacturer's manual (Plex Flex Single Set, Becton Dickinson, USA). The samples were read in an LSR II flow cytometer using FACS Diva software (Becton Dickinson, USA). Prior to reading, the cytometer was calibrated using the calibration beads included in the test pack (Cytometer Setup Beads). Based on the FSS/SS images, the beads were gated and fluorescence was read. The analysis was performed using an FCAP Array software (Becton Dickinson, USA).

### 2.3. Statistical Analysis

The data were screened for obvious data entry errors, missing values, and outliers. Arithmetical means and standard deviations or 95% CI were used to describe continuous variables, whereas proportions were used for categorical variables. A multivariate logistic regression model was fitted to the data. During the model building phase first-order interactions between the covariates were assessed. Only the interaction between serum TGF-*β*1 concentrations and the duration of diabetes proved to be statistically significant and was included into the final model. We assessed the marginal effects to improve the interpretation of the interaction of these two continuous covariates. The level of statistical significance was set at 0.05. All the statistical analyses were carried out using STATA 11.0 (StataCorp, Texas, USA) statistical package.

## 3. Results

### 3.1. Clinical Characteristics of Subjects with T1DM

A total of 116 children and adolescents with T1DM were examined. Patients with T1DM were 13.3 ± 3.9 years old with the duration of diabetes of 9.7 ± 3.6 years, mean arterial blood pressure of 86.0 ± 8.0 mmHg, serum levels of HbA1c 8.5 ± 1.9%, and albumin excretion rate of 24 ± 19 mg/24 h. The clinical and biochemical characteristics of patients with T1DM are shown in Table 1.

### 3.2. Serum Levels of TGF-*β*1 in T1DM Patients and Healthy Controls

**Figure 1:**
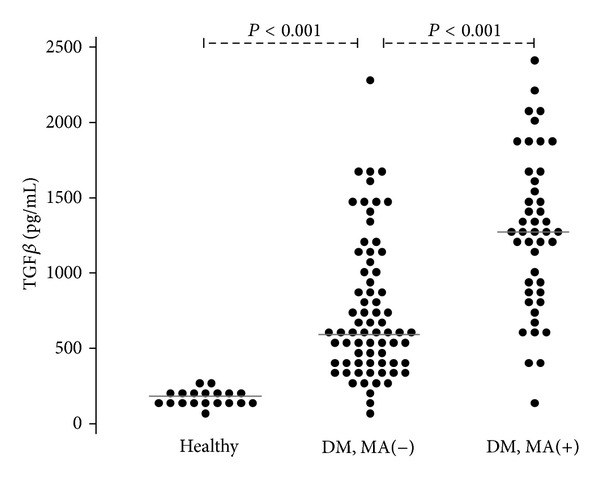
Serum concentrations of TGF-*β*1 in T1DM patients and healthy controls.

The patients with T1DM and the presence of microangiopathy (MA+) displayed statistically significant higher serum levels of TGF-*β*1 (*P* < 0.001) as compared to the patients with T1DM but without microangiopathy (MA−). However, the patients with T1DM but without microangiopathy had significantly higher serum TGF-*β*1 concentrations compared to the control subjects (*P* < 0.001) (Figure 1).

In the patients with T1DM the relative frequency of microangiopathy was 38.8% (45 cases, 95% CI: 29.8%–47.8%). 

In order to study the association between the duration of T1DM and the relevant variables we divided the former into four consecutive intervals. These intervals and data related to the frequency of microangiopathy are shown in Table 2. We found 11 cases of isolated nephropathy, 8 cases of isolated retinopathy, and 26 cases where both nephro- and retinopathy were present.

**Figure 2:**
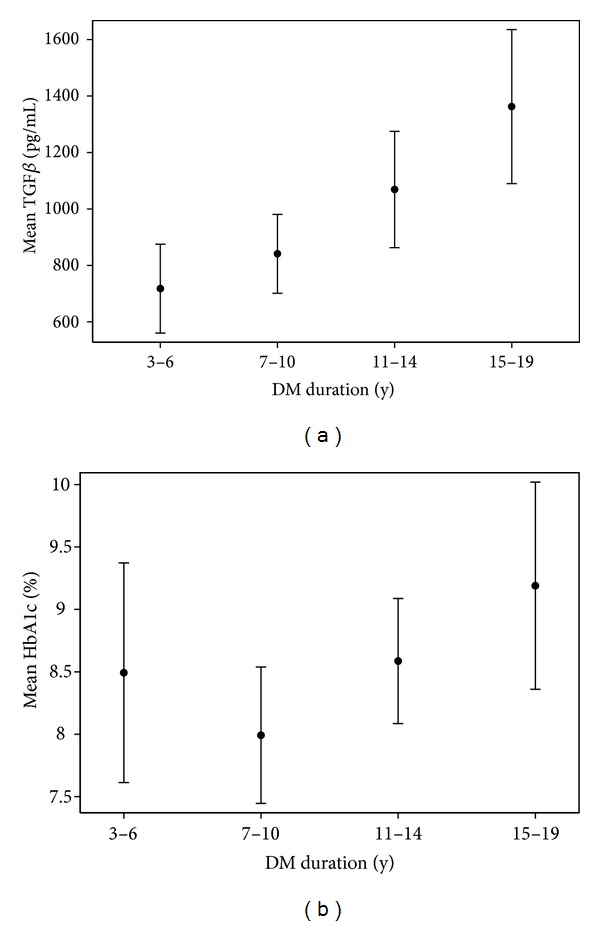
Association between duration of T1DM, TGF-*β*1, and mean HbA1c Whiskers indicates 95% CIs.

We noted a significant relationship between the duration of T1DM and the concentrations of the TGF-*β*1, but the HbA1c levels did not differ significantly between the subgroups of diabetes duration (Table 3, Figures 2(a) and 2(b)).

### 3.3. The Multivariate Logistic Regression Model

An initial multivariate logistic regression model was fitted to the data with all the relevant risk factors except for TGF-*β*1. The log-likelihood value for this model was recorded. Then, serum TGF-*β*1 concentrations were added to the model. First-order interactions between serum TGF-*β*1 levels and the other predictors were assessed. Only the interaction between serum TGF-*β*1 concentrations and the duration of diabetes proved to be statistically significant and was included into the final model (Table 4). The final model showed to fit the data significantly better compared to the initial model (*χ* ^2^(2) = 11.5, *P* = 0.003).

**Figure 3:**
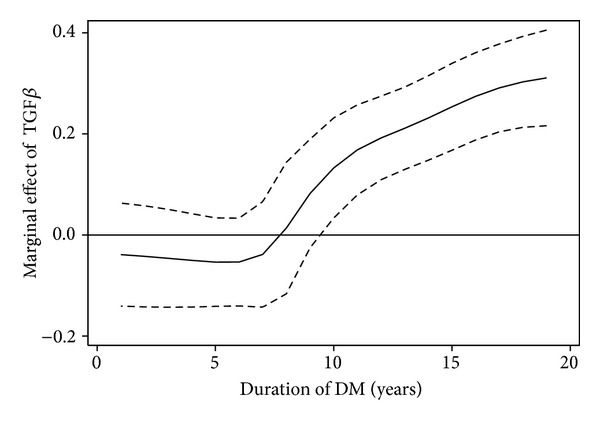
The effect of TGF-*β*1 on probability of microangiopathy depending on the duration of T1DM. The dashed lines indicate the 95% confidence interval for the effect.

In order to estimate how TGF-*β*1 influenced the occurrence of microangiopathy, given the significant interaction with the duration of DM, we analyzed the marginal effect. We noted that the increase of TGF-*β*1 was associated with a significant increase of the probability of microangiopathy but after the first 10 years of DM. In the following years of T1DM duration this effect tended to be stronger (Figure 3). For example, in the 15th year of DM, a 1 SD increase in TGF-*β*1 concentration was associated with a .25 (i.e., 25%) increase in the probability of microangiopathy when remaining predictors were held constant. 

## 4. **Discussion**

Previous studies have shown that the development of vascular changes in the eyes and kidneys of patients with T1DM result from many interrelated factors. Apart from well-known biochemical and hemodynamic factors, particular attention has been drawn in T1DM patients to the involvement of inflammatory factors through the production of cytokines, chemokines, and growth factors [4, 7, 24–27]. Despite the engagement of many research centers, the mechanisms leading to the development of diabetic microangiopathy in children and adolescents with T1DM still have not been fully elucidated. That is why further research is needed to solve the pending problem of chronic vascular complications in T1DM patients. 

In the present study we have tried to evaluate the relationship between serum TGF-*β*1 levels and the duration of T1DM in children and adolescents. We have found significantly higher serum TGF-*β*1 concentrations in children and adolescents with T1DM and microangiopathy as compared with patients with T1DM but no signs and symptoms of diabetic microangiopathy. However, we have found significantly higher serum TGF-*β*1 in a group of patients with T1DM but without microangiopathy as compared with the healthy control subjects. Already at this stage of the study it was found that there is an increase of serum TGF-*β*1 concentrations depending on the severity of microvascular complications in children and adolescents with T1DM. In addition, we detected that the longer the duration of diabetes, the higher the concentration of TGF-*β*1 in children and adolescents with T1DM. Similarly, the incidence of vascular complications was increasing over time. The multivariate logistic regression model and the assessment of the marginal effect allowed to establish a relationship between various factors and the probability of microangiopathy. The interaction between serum TGF-*β*1 concentrations and the duration of diabetes proved to be statistically significant and was included into the final model. We have shown that the increase of TGF-*β*1 concentrations was an independent factor aggravating the likelihood of microvascular complications in our study group of children and adolescents with T1DM. This effect was statistically significant in the patients with diabetes of at least 10 years duration. In the successive years of the disease, a further and even stronger effect of TGF-*β*1 on the development and progression of diabetic microangiopathy was observed. For example, in the fifteenth year of diabetes duration, an increase in the serum TGF-*β*1 concentrations by 1 SD was associated with a 25% increase in the probability of microangiopathy when the remaining predictors were held constant. What is more, in our recent study we established the limit for TGF-*β*1 concentrations on the presence of diabetic retinopathy in children and adolescents with T1DM [28]. We suggest that in patients with T1DM, TGF-*β*1 levels may correlate with the degree of eye and kidney damage. Similar results were obtained in the patients with T1DM of over 10-year duration [29]. A statistical correlation between diabetes duration and the severity of morphological changes in kidney biopsy has been shown. Other researchers have shown higher intravitreal TGF-*β*1 concentrations in adult T1DM patients with PDR, which in turn may be associated with retinal angiogenesis and tissue fibrosis at the vitreoretinal interface [30]. In summary, the results of our study indicate that serum TGF-*β*1 levels are one of the factors that may have an impact on the progression of vascular complications in children and adolescents with T1DM. 

## Figures and Tables

**Table 1:** Clinical and laboratory characteristics of patients.

Parameter	Value	Range
Age (y)	13.3 ± 3.9	4–20
Duration of T1DM (y)	9.7 ± 3.6	3–19
Mean arterial blood pressure (mmHg)	86.0 ± 8.0	65–105
Serum HbA1c (%)	8.5 ± 1.9	5.8–14.3
Albumin excretion rate (mg/24 h)	24 ± 19	0.20–120

Data are given as a number of subjects (%) or mean ± SD.

**Table 2:** The number of patients with T1DM and microangiopathy depending on the disease duration.

DM duration	Microangiopathy					Total
	Absent	Present	N	R	N + R	
3–6 years	26	2	1	1	0	28
7–10 years	31	9	6	2	1	40
11–14 years	12	17	3	1	13	29
15–19 years	2	17	1	4	12	19

Total	71	45	11	8	26	116

Abbreviations: N: isolated nephropathy, R: isolated retinopathy, N + R: combined nephro- and retinopathy.

**Table 3:** Means and standard deviations of HbA1c and TGF-*β*1 over four consecutive DM duration intervals.

DM duration	HbA1c (%)	TGF-*β*1 (pg/mL)
3–6 years	8.5 ± 2.4	718 ± 425
7–10 years	8.0 ± 1.8	841 ± 451
11–14 years	8.6 ± 1.4	1069 ± 566
15–19 years	9.2 ± 1.8	1362 ± 607

**Table 4:** The final logistic regression model for microangiopathy.

Predictor	*β*	−95% CI	+95% CI	*P *
Female	2.81	0.83	4.78	0.005
MAP	1.65	0.03	0.30	0.015
Age	−0.10	−0.34	0.13	0.396
HbA1C	0.30	−0.16	0.77	0.199
TGF-*β*1	−3.83	−7.44	−0.23	0.037
DOD	0.75	0.35	1.14	0.000
TGF-*β*1 × DOD	0.49	0.10	0.88	0.013
Intercept	−10.9	−16.0	−5.7	0.000

Abbreviations: *β*: unstandardized regression coefficient, MAP: mean arterial pressure, DOD: duration of T1DM, TGF-*β*1 × DOD: interaction between TGF-*β*1 and DOD.
